# Computational analysis of the functional impact of MHC-II-expressing triple-negative breast cancer

**DOI:** 10.3389/fimmu.2024.1497251

**Published:** 2024-11-27

**Authors:** Yang Cui, Weihang Zhang, Xin Zeng, Yitao Yang, Sung-Joon Park, Kenta Nakai

**Affiliations:** ^1^ Department of Computational Biology and Medical Sciences, Graduate School of Frontier Sciences, University of Tokyo, Tokyo, Japan; ^2^ Human Genome Center, Institute of Medical Science, University of Tokyo, Tokyo, Japan

**Keywords:** breast cancer, machine learning, MHC-II pathway, multi-omics data integration, tumor microenvironment

## Abstract

The tumor microenvironment (TME) plays a crucial role in tumor progression and immunoregulation. Major histocompatibility complex class II (MHC-II) is essential for immune surveillance within the TME. While MHC-II genes are typically expressed by professional antigen-presenting cells, they are also expressed in tumor cells, potentially facilitating antitumor immune responses. To understand the role of MHC-II-expressing tumor cells, we analyzed triple-negative breast cancer (TNBC), an aggressive subtype with poor prognosis and limited treatment options, using public bulk RNA-seq, single-cell RNA-seq, and spatial transcriptomics datasets. Our analysis revealed a distinct tumor subpopulation that upregulates MHC-II genes and actively interacts with immune cells. We implicated that this subpopulation is preferentially present in proximity to regions in immune infiltration of TNBC patient cohorts with a better prognosis, suggesting the functional importance of MHC-II-expressing tumor cells in modulating the immune landscape and influencing patient survival outcomes. Remarkably, we identified a prognostic signature comprising 40 significant genes in the MHC-II-expressing tumors in which machine leaning models with the signature successfully predicted patient survival outcomes and the degree of immune infiltration. This study advances our understanding of the immunological basis of cancer progression and suggests promising new directions for therapeutic strategies.

## 1 Introduction

Triple-negative breast cancer (TNBC) is an aggressive subtype of breast cancer, constituting 10%–20% of all breast cancer cases. Characterized by the absence of estrogen receptor and progesterone receptor as well as the human epidermal growth factor receptor 2 (HER2) receptor, TNBC is notable for its invasive nature and poorest prognosis (1). Additionally, TNBC does not respond to existing endocrine and HER2-targeted therapies, leading to challenges in clinical treatment strategies. The tumor microenvironment (TME) in TNBC plays a critical role in immunoregulation and tumor progression (2). Within the TME, the major histocompatibility complex class II (MHC-II) pathway is a crucial regulator for immune surveillance: MHC-II genes activate CD4+ helper T cells by presenting antigens that facilitate effective immune responses (3), and the CD4+ helper T cells activate CD8+ cytotoxic T cells eliminating tumor cells through a sustained and effective memory response (4–8).

The constitutive process of antigens mediated by the MHC-II pathway is typically restricted to professional antigen-presenting cells (APCs), such as dendritic cells, macrophages, and B cells. On the other hand, several studies have shown that MHC-II genes are also expressed in tumor cells. This expression enhances tumor recognition by the immune system, which is thought to increase immune infiltration and a favorable prognosis (3, 9, 10). However, it remains unclear whether the expression of MHC-II genes originates from tumor cells or immune cells, as previous studies have primarily analyzed bulk RNA-seq cohorts (9, 10). While methods like immunohistochemistry or immunofluorescence can help address this issue, these methods are limited in detecting a comprehensive array of proteins and may not fully differentiate the source of MHC-II expression (3, 10).

In this study, we conducted multiomics data analysis to computationally decompose tumor and immune cells within the TNBC microenvironment, aiming to elucidate molecular signatures associated with the MHC-II-expressing tumors. We then used these molecular signatures to predict clinical survival outcomes and levels of immune infiltration. Our findings provide insights into the functional significance of the TME in TNBC subtypes that are linked to improved patient survival.

## 2 Results

### 2.1 Identifying TNBC subtypes in large cohorts

**Figure 1:**
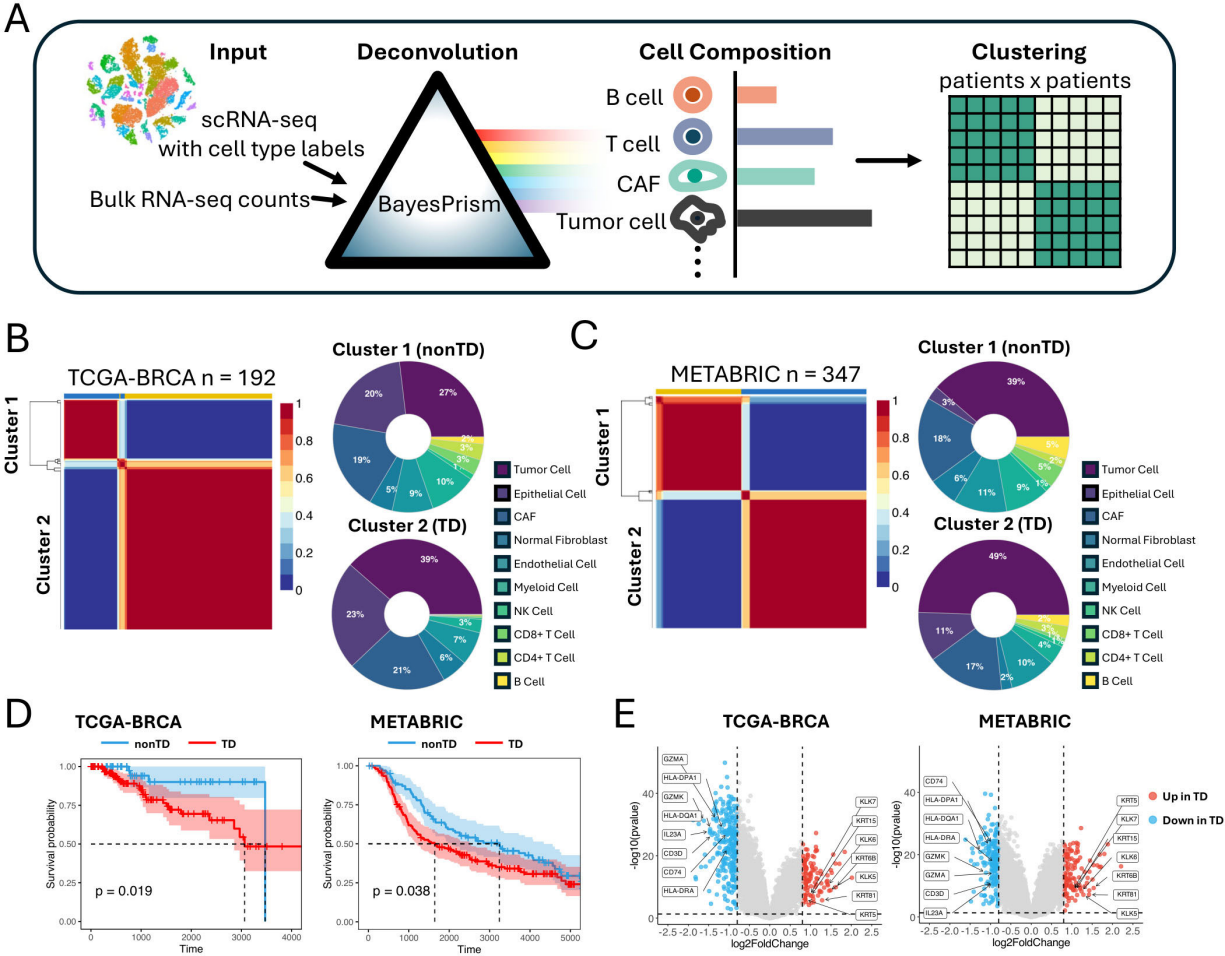
Identifying TNBC patient groups based on cell composition in the TME. **(A)** Schematic representation of the workflow deconvoluting cell compositions and clustering the patients. **(B)** NMF clustering of TCGA-BRCA cohorts based on the cell compositions. Donut plot showing the cell composition of each cluster. **(C)** NMF clustering of METABRIC cohorts based on the cell compositions. Donut plot showing the cell composition of each cluster. **(D)** Kaplan–Meier plot showing the worse clinical outcome in TD patient cluster in the TCGA-BRCA and METABRIC cohorts (log rank test, P < 0.05). **(E)** Volcano plot showing differentially expressed genes between TD and nonTD patient clusters in TCGA-BRCA and METABRIC cohorts. Red dots and blue dots represent significantly upregulated and downregulated differentially expressed genes respectively (threshold: |log2FC| > 1, P < 0.05). TNBC, triple-negative breast cancer; TME, tumor microenvironment; NMF, non-negative matrix factorization; TD, tumor dense; nonTD, non- tumor dense; FC, fold change.

We designed a computational pipeline to cluster TNBC patients based on cellular compositions in TNBC TME (**Figure 1A**) by collecting 539 bulk RNA-seq datasets from TCGA-BRCA and METABRIC cohorts (11). To annotate cell types and determine their proportions within these datasets, we first created reference cell types using scATOMIC (12) with seven scRNA-seq datasets of TNBC patients (13) (**Supplementary Figure S1A**). Subsequently, we employed BayesPrism (14) to deconvolute the cellular compositions in the cohort datasets based on these reference cell types (**Supplementary Figure S1B**).

Applying non-negative matrix factorization (NMF) (15) to the cellular composition datasets by optimizing its hyperparameters (**Supplementary Figure S1C**), we identified two patient clusters from each of the cohorts characterized by varying proportions of tumor cells and immune cells (**Figures 1B, C**). Cluster 2, characterized by a higher proportion of tumor cells and fewer immune cells, is hereafter referred to as tumor dense (TD) patient cluster, whereas cluster 1 is referred to as nonTD patient cluster. This pattern remained consistent, even when merging corresponding clusters from both cohorts (**Supplementary Figure S1D**). Remarkably, these patient groups exhibited distinct survival outcomes, indicating a relationship between the cellular composition and aggressive malignancy (**Figure 1D**).

Comparing the bulk transcriptome profiles of the TD and nonTD patients (**Figure 1E**; **Supplementary Figure S1E**), the TD patients exhibited upregulation of keratins (*KRT81*, *KRT6B*, *KRT15*, *KRT5*) and kallikreins (*KLK5*, *KLK6*, *KLK7*), indicating active extracellular matrix (ECM) remodeling and tumor expansion (16–18). In contrast, the nonTD patients showed upregulation of immune-related genes, such as HLA class II antigens (*HLA-DRA*, *HLA-DPA1*, *HLA-DQA1*), *CD74*, and genes related to cytotoxic and helper T- cell activities (*GZMK*, *GZMA*, *CD3D*, *IL23A*), suggesting active antigen processing (19–21). Gene Ontology (GO) analysis further highlighted that the upregulated genes in each patient cluster are highly involved in crucial biological processes: epidermis development and extracellular matrix organization in TD-upregulated genes, and immune activity and MHC-II arrangement in nonTD-upregulated genes (**Supplementary Figure S1F**).

Collectively, our results underscored distinct subtypes of TNBC patients characterized by significant alterations in gene expression relevant to immune and metastatic potential. These characteristics were identified by grouping patients based on cell-type composition that indicated their influence on survival outcomes.

### 2.2 Characterizing TNBC subtypes by single-cell data

**Figure 2:**
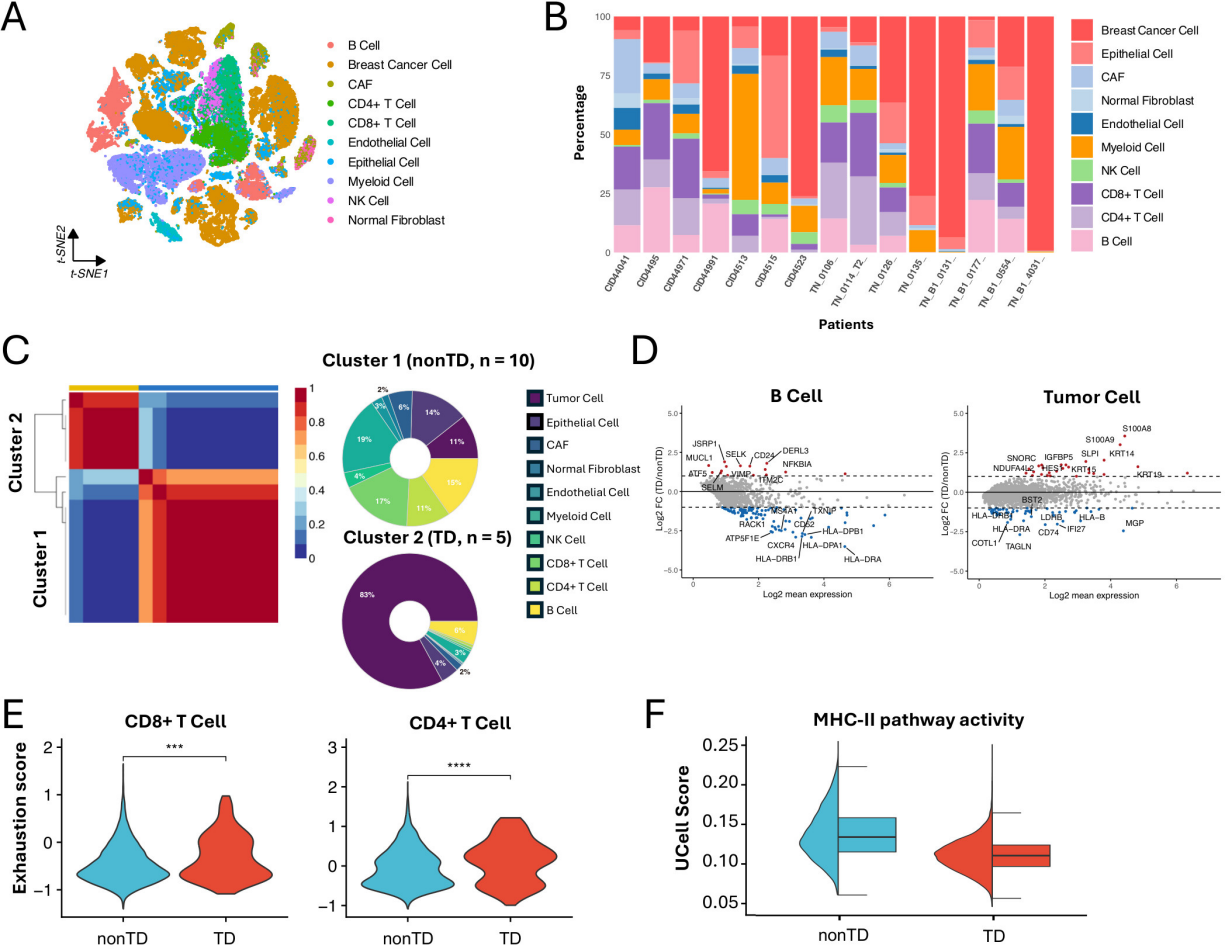
Characterizing TNBC subtypes in single-cell datasets. **(A)** t-SNE visualization of 65,496 cells from 15 TNBC patients analyzed by scRNA-seq. **(B)** Bar plot showing the cell proportions of each patient from the scRNA-seq dataset. **(C)** NMF clustering of the scRNA-seq dataset base on the cell composition. Donut plot showing the cell composition of each cluster. **(D)** MA plot showing differentially expressed genes between TD and nonTD B cells and tumor cells (|log2FC| > 1, P < 0.05). **(E)** Violin plot showing the exhaustion score of CD4+ T cell and CD8+ T cell in TD and nonTD (two-sided Wilcoxon test, ∗∗∗P < 0.001, ∗∗∗∗P < 0.0001). **(F)** Half violin plot showing MHC-II pathway activity scores in TD and nonTD tumor cells.

To inspect the TD and nonTD patient clusters at a refined level, we collected 15 scRNA-seq datasets of TNBC patients (13, 22), comprising a total 65,496 cells for further analysis. To ensure proper integration of scRNA-seq data from different sources, we evaluated three batch correction tools to identify the most suitable method (**Supplementary Figure S2A**). The *k*-nearest-neighbor batch-effect test (kBET) (23) was used to quantify the batch effect and assess the performance of these tools. Among CCA (24), MNN (25), and Harmony (26), Harmony demonstrated the best performance (**Supplementary Figure S2B**). Therefore, we applied Harmony to effectively remove batch effects. Unlike the cohort analysis requiring the deconvolution of cellular compositions, we directly derived the cell type counts for each of the 15 patients by annotating the single-cell population by scATOMIC (**Figures 2A, B**). Then, we applied the optimized NMF (**Supplementary Figure S2C**) that identified two patient clusters corresponding to the TD and nonTD characteristics (**Figure 2C**).

Consistent with the characteristics observed in the cohort analysis, the immune-related and tumor cells markedly varying in abundance exhibited different expression patterns of essential genes between the clusters. For instance, differentially expressed gene (DEG) analysis revealed that B cells and tumor cells in the nonTD cluster exhibit upregulation of marker genes in MHC-II pathway (e.g., *HLA-DRB* and *HLA-DRA*), whereas tumor cells in the TD cluster show upregulation of keratins (**Figure 2D**), supported by corresponding GO biology pathway (BP) term enrichments (**Supplementary Figure S2D**). Additionally, CD4+ and CD8+ T cells in the TD cluster demonstrated higher levels of exhaustion (**Figure 2E**), suggesting decreased T- cell functionality and reduced efficacy in tumor elimination (27).

Furthermore, we assessed the degree of MHC-II pathway activity in the tumor cell population of each patient cluster by calculating activity scores with Ucell (28). This analysis involved standardizing the average expression levels of a relevant gene set in scRNA-seq data. The gene set of the MHC-II pathway was obtained from the Molecular Signatures Database (MSigDB) (29, 30). The result revealed significant activation of the MHC-II pathway in tumor cells from nonTD patients (**Figure 2F**). This finding was supported by the expression profiles of MHC-II pathway marker genes in individual tumor cells (**Supplementary Figure S2E**).

Taken together, the TNBC subtypes identified through single-cell analysis were distinguished by variations in cell population abundance and their functional characteristics. These findings align closely with those from the bulk RNA-seq data analysis. Notably, we observed that tumor cells in nonTD patients activate MHC-II-related genes, which may contribute to the improved survival outcomes seen in the cohort analysis.

### 2.3 Identifying tumor cells expressing MHC-II genes

**Figure 3:**
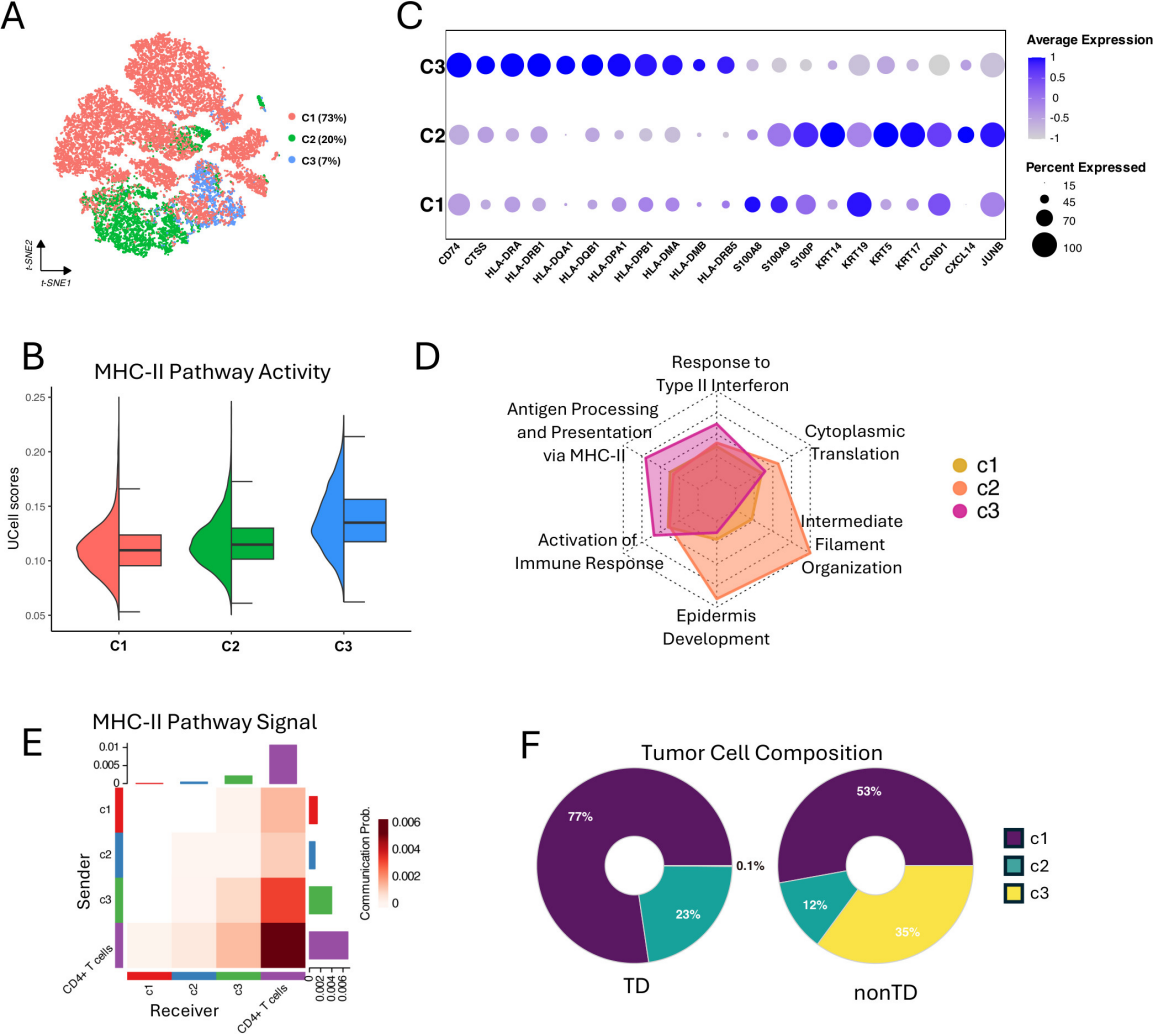
Characterizing of MHC-II expressing tumor cells. **(A)** t-SNE visualization showing three clusters (C1, C2, and C3) of 23,186 tumor cells. **(B)** Half violin plot showing MHC-II pathway activity scores in each tumor cell cluster. **(C)** Bubble plot showing marker gene expression profile of each tumor cell cluster. Bubble size indicates the expression percentage of each tumor cell cluster, and the color shows the average expression level. **(D)** Radar plot showing the GO enrichment analysis results of biological processes for differentially expressed genes in the three clusters, using gene ratio of biological processes terms for each tumor cell cluster. **(E)** Heatmap showing the MHC-II pathway signaling interactions between the tumor cell clusters and CD4+ T cells. X-axis represents the receiver and Y-axis represents the sender. **(F)** Donut plot showing the proportions of C1, C2, and C3 tumor cells in TD and nonTD.

To explore the relationship between tumor cell heterogeneity and MHC-II activity, we first isolated 23,186 tumor cells from **Figure 2A** and corrected batch effects using Harmony. Since directly performing NMF on the expression data of 23,186 tumor cells was extremely time-consuming and computationally intensive, we applied the SuperCell method (31) for dimensionality reduction prior to clustering. As a result, we identified 580 tumor metacells. Metacell is a feature aggregated by grouping highly similar single cells, reducing the complexity of the data while retaining important biological information (31). Subsequently, we performed principal component analysis (PCA) on the metacell features and applied the optimized NMF using the principal components (n = 50) (**Supplementary Figure S3A**). This approach identified three metacell clusters; C1, C2, and C3 (**Supplementary Figure S3B**). Finally, we annotated the original tumor cells based on these metacell clusters (**Figure 3A**) for downstream analyses.

Interestingly, the C3 cells, 7% in the total tumor cells, exhibited a higher degree of MHC-II pathway activity, as indicated by UCell scores (**Figure 3B**) and the elevated expression of key MHC-II-related genes in DEG analysis result (**Supplementary Figure S3C**). In particular, HLA class II antigens were markedly expressed in the C3 tumor cells (**Figure 3C**). Additionally, comparing the GO-BP enrichments with the upregulated genes in each cluster revealed that the genes in C3 are notably associated with antigen processing and presentation via MHC-II, response to type II interferon, and activation of immune response (**Figure 3D**; **Supplementary Figure S3D**). This suggests that the activation of the MHC-II pathway, likely driven by IFN-γ stimulation, may contribute to the immunogenicity of the C3 tumors (3).

To infer the interplay of each tumor cluster with immune cells, we quantified the communication probabilities between tumor clusters and CD4+ T cells, focusing on the MHC-II-mediated interactions, using CellChat (32). This analysis evaluates the total links of outgoing (sender) and incoming (receiver) signaling within a network constructed from ligand–receptor pairs found in a given single-cell group. The result showed that C3 has the highest level of MHC-II interaction with CD4+ T cells (**Figure 3E**), suggesting that the C3 tumor cells are particularly important for robust antitumor responses. We also investigated the interactions between tumor clusters and other components within the TME. We further found that C1 tumor cells exhibit strong interactions with cancer-associated fibroblasts (CAF) via the COLLAGEN pathway, actively release VEGF signals, and significantly engage in NOTCH signaling (**Supplementary Figure S3E**). These findings suggest that C1 tumor cells are characterized by enhanced ECM remodeling, greater invasiveness, and higher tumor stemness, consistent with our GO-BP enrichment analysis results (33–35). Additionally, we observed that the C1, C2, and C3 all received IFN-II signals, even C1 and C2 showing stronger signals than C3 (**Supplementary Figure S3E**). However, only C3 demonstrated the response of the IFN-γ stimulation and activation of the MHC-II pathway, highlighting C3’s unique sensitivity to the IFN-γ.

Collectively, our findings highlight the heterogeneous tumor cell population in the TNBC TME. We successfully identified the subpopulation of TNBC tumor cells, C3, that express MHC-II genes and actively interact with immune cells and sensitivity to IFN-γ, which suggests its functional importance in repressing tumor progression. Indeed, as shown in **Figure 3F**, the C3 cells were predominantly found in the nonTD patients defined in **Figure 2C**, rather than in the TD patients who exhibit impaired immune function.

### 2.4 Inferring the important genes in the MHC-II-expressed tumor cells

Given the significance of C3 tumor cells identified through single-cell analysis, we aimed to capture prognostic signatures based on the C3 marker genes. Firstly, we annotated cell types, including C1, C2, and C3, in the scRNA-seq data (13, 22) using scATOMIC. Next, we merged cell types with similar expression profiles for improving BayesPrism’s ability to extract features for C3 cells. Following this, we profiled gene features for these cell types using BayesPrism and detected 793 marker genes for C3 (**Supplementary Figure S4**). Combined with 60 genes differentially upregulated in C3 compared with other tumor cells (**Supplementary Figure S3C**), we used the 853 genes for analyzing prognostic signatures. To assess the prognostic potential of these C3 marker genes, we performed univariate Cox regression analysis on the genes to identify those significantly associated with patient survival outcomes. Subsequently, we applied multivariate Cox regression analysis to refine the prognostic signature by considering potential confounding factors and interactions between genes.

**Figure 4:**
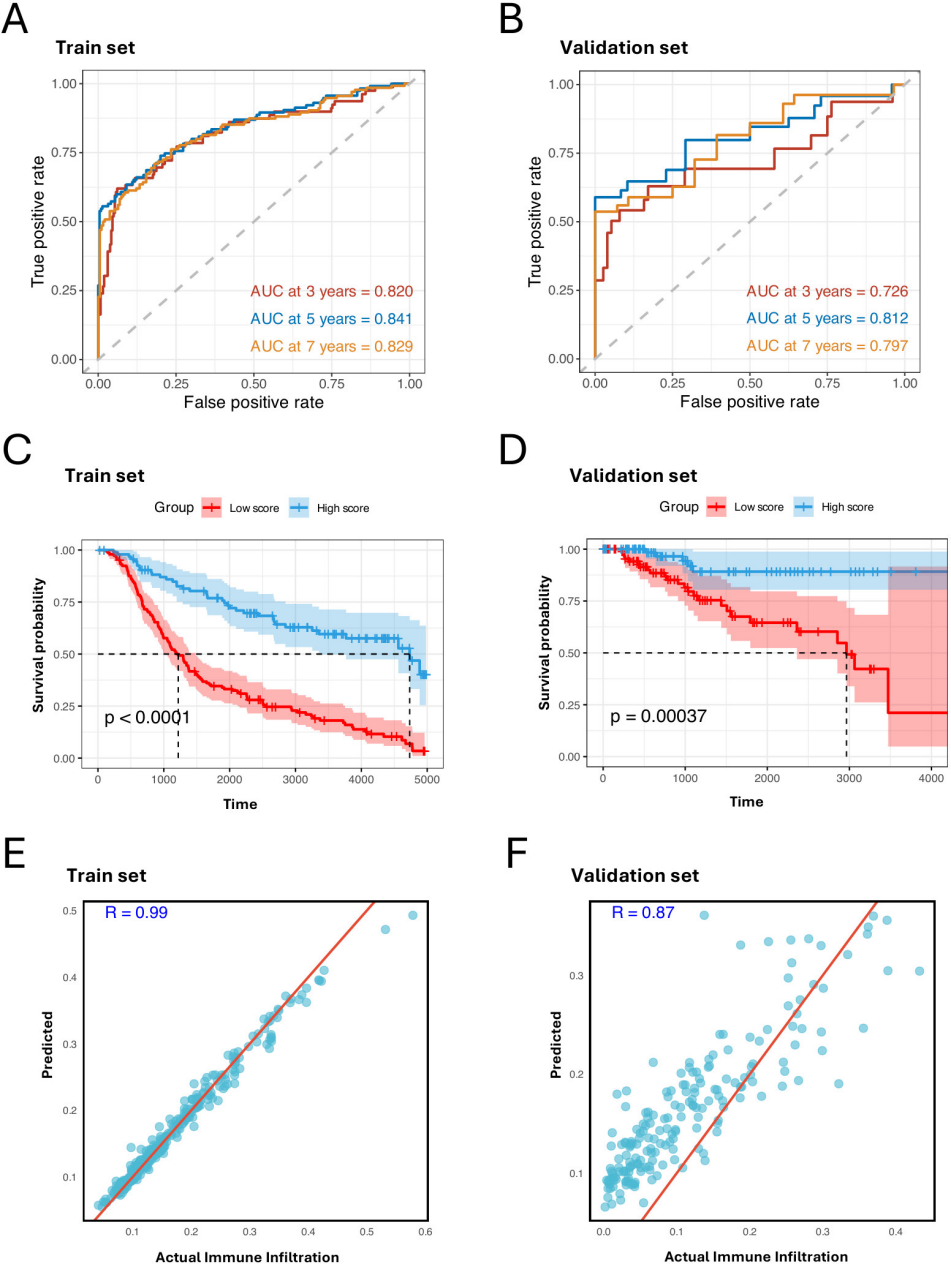
MHC-II-expressed tumor cell marker genes predict prognosis and immune infiltration. **(A, B)** ROC curves of the prognostic signature for predicting the risk of death at 3, 5, and 7 years in train set, test set, and validation set. **(C, D)** Kaplan–Meier plot showing better prognosis in patients with high signature score in the train set, test set, and validation set. The high score group and low score group are identified by the mean score of the signature (log- rank test, P < 0.001). **(E, F)** Scatter plots showing the Pearson correlation between predicted and actual immune cell infiltration levels in the train set, test set, and validation set. X-axis represents the actual immune cell infiltration level, and Y-axis represents the predicted immune cell infiltration level.

For the multivariate Cox regression analysis, we employed 10-fold cross-validation on the METABRIC cohort to train the model. The cohort was randomly split into training and test sets in each fold, and the TCGA-BRCA cohort was used as an independent validation set. This analysis identified a prognostic signature consisting of 40 genes and their coefficients from the cross-validated training set (**Supplementary Table S1**). This signature demonstrated high predictive capability with an area under the curve (AUC) of 0.820 at 3 years, 0.841 at 5 years, and 0.829 at 7 years (**Figure 4A**). It also achieved remarkable AUCs in the validation set (**Figure 4B**). Among the genes in the prognostic signature, *NME7* and *GPX1* stood out with the highest coefficients: *NME7* has the highest positive coefficient, and *GPX1* has the highest negative coefficient. This underscores their crucial roles in the prognostic characterization of C3 tumor cells. Notably, the upregulation of *NME7* is known to improve survival outcomes and function in tumor suppression (36). On the other hand, *GPX1*, which has been identified highly expressed in TNBC cell lines, plays a key role in cell adhesion and spreading by modulating FAK/c-Src activation. The depletion of *GPX1* has been shown to impair TNBC metastasis processes, further highlighting its importance (37).

After calculating the signature score for each patient by multiplying the 40 gene expression levels by their corresponding coefficients, we divided the patients into two groups based on whether their scores were above or below the median. Notably, the patients with higher signature scores exhibited better prognoses (**Figures 4C, D**). To further confirm the impact of the C3 signature, we employed a random forest model using the 40 genes to predict immune infiltration levels: immune cell infiltration was estimated based on the relative abundance of immune cells annotated in **Supplementary Figure S1D**. We observed strong positive correlations between the predicted and observed immune infiltration levels (**Figures 4E, F**), supporting that C3 tumor cells significantly impact immune biology within TBNC TME.

Our findings highlight that the prognostic signature with the 40 genes captured from the C3 tumor cells effectively distinguishes clinical outcomes and reliably estimates immune infiltration. This suggests that these genes may serve as potential therapeutic targets and provide valuable insights into the immune landscape associated with TNBC progression and response.

### 2.5 Spatial localization of the MHC-II-expressed tumor cells

**Figure 5:**
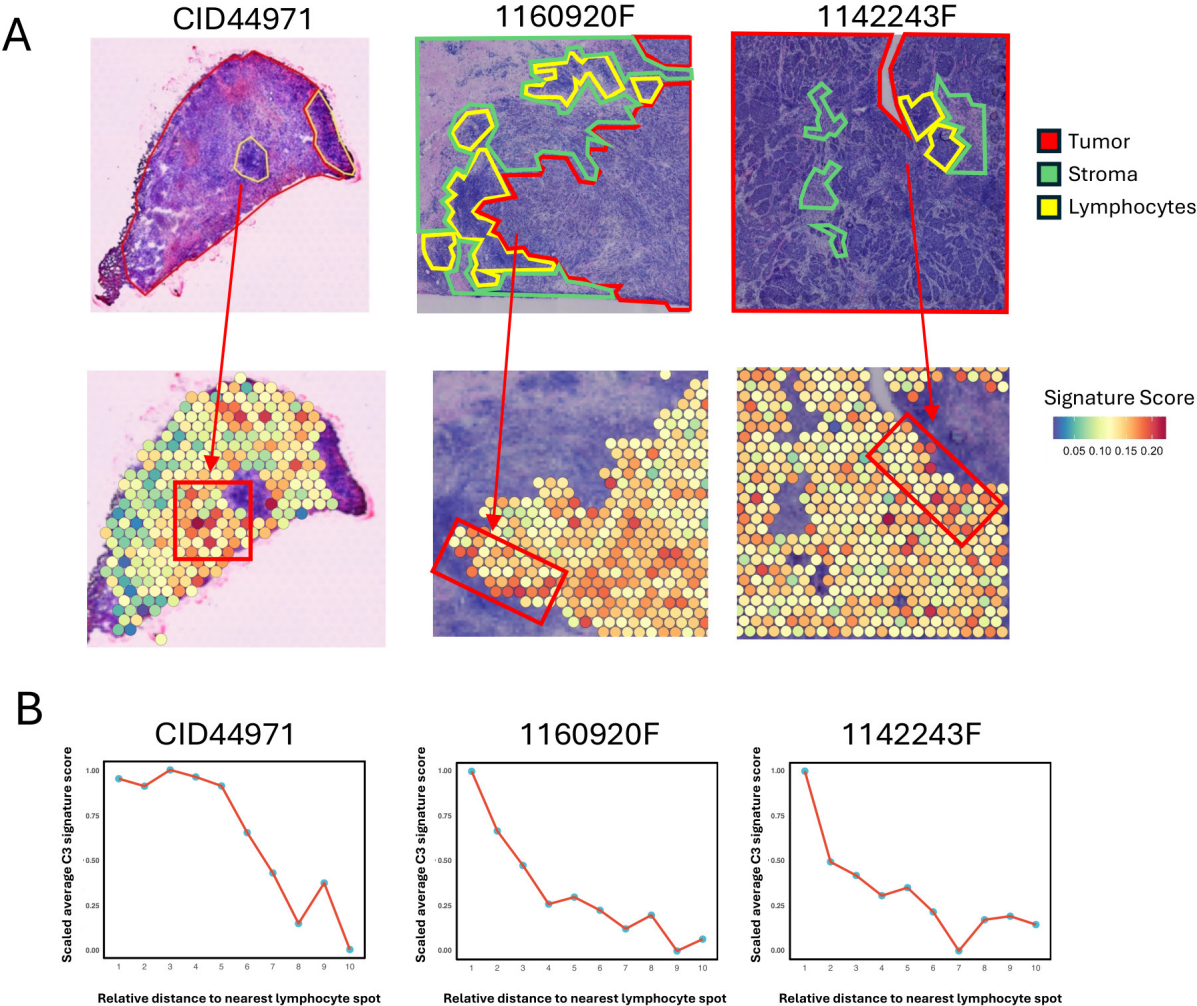
Spatial co-localization of MHC-II expressing tumor cells with immune infiltration regions. **(A)** Spatial visualization of the C3 tumor cell signature score in tumor regions of TNBC tissue sections. The top panels show three TNBC samples (CID44971, 1160920F, 1142243F) with tumor areas outlined in red, stroma in green, and lymphocyte regions in yellow. The bottom panels display the C3 tumor cell signature score of each spot in tumor regions. The arrow indicates the tumor area proximal to the lymphocyte spot. **(B)** Line plots showing the trend of scaled average C3 tumor cell signature score of tumor spot for the Euclidean distance to the nearest lymphocyte spot. For each spatial transcriptomic data, tumor spots are assigned to 10 bins by their Euclidean distance to the nearest lymphocyte spot, and then average C3 tumor cell signature score of tumor spots within each bin is calculated and scaled for comparison.

To investigate the spatial implications of C3 tumor cells in the TNBC microenvironment, we analyzed the spatial transcriptomic (ST) data, which includes manually annotated regional labels (13). Given that ST spots may contain multiple cell types, we calculated the signature score of the 40 C3 marker genes for each spot using the UCell method. Of note, lymphocytes within tumor tissue are the central regions for immune infiltration (38). We observed that spots with higher signature scores were predominantly located near lymphocyte areas (**Figure 5A**). This pattern was further supported by quantitative analysis that showed a decrease in signature scores with increasing Euclidean distance from the nearest lymphocyte (**Figure 5B**; **Supplementary Figures S5A, B**).

These findings revealed spatial shapes of the immune landscape within the TNBC TME, conferring by the association of MHC-II-expressed tumor cells and immune infiltration.

## 3 Discussion

We aimed to characterize tumor cell subtypes that express MHC-II genes in the TNBC microenvironment. Although the significance of this tumor type has been recognized (3, 9, 10), understanding the underlying molecular mechanisms has been challenging due to tumor cell heterogeneity. To address this, we performed a comprehensive single-cell analysis and identified a distinct subpopulation of tumor cells, termed C3. Despite constituting only approximately 6% of the total tumor cells, this population exhibited upregulation of MHC-II genes and actively interacted with immune cells, such as CD4+ T cells, prevalent in TNBC patients who have better survival outcomes, i.e., the nonTD patient group. The presence of this minority subpopulation suggests that C3 cells may represent a specialized subset with a unique and efficient role in immune modulation, contributing disproportionately to the immune response and better survival outcomes.

These observations underscore the key role of MHC-II genes in presenting tumor antigens, which are crucial for activating the CD4+ and CD8+ T- cell responses (3–8). Traditionally, these antigens are presented to the immune cells by professional APCs (3). However, our findings revealed a distinct pathway of immune modulation, extending the recent studies (9, 10), where tumor-derived antigens are processed by the C3 tumor cells localized in proximity to regions in immune infiltration.

The expression of MHC-II is driven by the transcriptional master regulator class II major histocompatibility complex transactivator (*CIITA*). *CIITA* is regulated by four distinct promoters: pI, pII, pIII, and pIV. Among these, pI and pIII have been shown to drive MHC-II expression in dendritic cells (DCs) and B cells, whereas the function of pII remains poorly understood (3, 39). In non-classical APCs, MHC-II expression is controlled by pIV, which can be induced by IFN-γ (40, 41). Our findings suggest that although tumor cells in the TME are exposed to IFN-γ, only the C3 tumor cells demonstrate a response to IFN-γ and the activation of the MHC-II pathway. In a study by Bo et al., partial or hemimethylation of the *CIITA* pIV promoter was shown to be sufficient to silence *CIITA* expression, leading to a loss of MHC-II expression (40). This loss of MHC-II could be reversed through treatment with hypomethylating agents. Based on this, we suggest that the heightened sensitivity of C3 tumor cells to IFN-γ and the activation of the MHC-II pathway may be regulated epigenetically.

It is important to consider the potential influence of genetic variation on the regulation of MHC-II gene expression. The MHC region is highly polymorphic, and this genetic diversity, particularly through cis-eQTLs, may modulate MHC-II expression in different tumor cells. Previous studies have identified cis-regulatory elements that control MHC-II transcriptional activity, such as the X, Y, and W/Z boxes. Variations within these elements could impact how tumor cells respond to immune signals such as IFN-γ and activate MHC-II expression (42). The heterogeneity observed in the activity of tumor cell subpopulations within the MHC-II pathway may be influenced by genetic variation in these regulatory elements. Future studies should incorporate genomic data, including SNP and eQTL analyses, to better understand the genetic factors affecting MHC-II expression in the TME. By accounting for these genetic factors, we may uncover additional layers of complexity that shape the immune landscape in TNBC and further clarify the role of MHC-II-expressing tumor cells in immune modulation.

Given the high immunogenicity of C3 tumor cells, we propose that C3 may contribute to enhanced immune infiltration within the TME. However, since MHC-II expression in tumor cells can also be induced by IFN-γ, there is a possibility that C3 represents a byproduct of a favorable immune microenvironment (3, 41). Challenging this view, studies have shown that in mouse tumor models transfected with *CIITA*, increased immune infiltration and tumor rejection were observed (43, 44). Additionally, depletion of DCs or macrophages did not affect the tumor rejection effect, demonstrating that MHC-II-expressing tumor cells can directly initiate antitumor immune responses and directly promote immune cell recruitment, rather than being merely a byproduct of a favorable immune microenvironment (45).

Furthermore, we identified a prognostic signature comprising 40 marker genes of C3, including *NME7* and *GPX1*, which show the highest positive and negative coefficients, respectively. Interestingly, *NME7* has been recognized for its tumor-suppressive role in breast cancer, whereas *GPX1* is linked to the regulation of tumor metastasis (36, 37). Additionally, *HCLS1*, the gene with the second highest positive coefficient in the prognostic signature, is known to positively correlate with immune infiltration in TNBC (46). These genes highlight the unique functional characteristics of the C3 tumor cells, emphasizing their potential role in modulating the tumor microenvironment. Our results clearly demonstrated that the combinatorial effect of these genes, in conjunction with the upregulation of MHC-II genes in TNBC, significantly explains patient survival outcomes and the degree of immune infiltration. However, there is still room for improvement in the performance of our signature. We plan to incorporate additional parameters, including multiomics data and further integration of molecular features in the future, to enhance the predictive accuracy.

In conclusion, our *in-silico* analysis highlights the significant role of MHC-II-expressing tumor cells as a key regulator of immune biology within the tumor microenvironment. This study advances our understanding of the immunological basis of cancer progression and suggests promising new directions for therapeutic strategies.

## 4 Materials and methods

### 4.1 Preparing transcriptomics data

The RNA-seq datasets were prepared from public databases and published papers. For bulk RNA sequencing data derived from TNBC patients, 192 samples in the TCGA-BRCA cohort (https://portal.gdc.cancer.gov/projects/TCGA-BRCA) and 347 samples in METABRIC (11) were prepared. For single-cell RNA-seq (scRNA-seq) data of TNBC patients, seven samples in GSE176078 and eight in GSE161529 were downloaded from GEO. Spatial transcriptomic datasets for three TNBC patients were obtained from Zenodo data repository (https://doi.org/10.5281/zenodo.4739739).

### 4.2 Processing single-cell RNA-seq data

scRNA-seq datasets GSE176078 and GSE161529 were processed using the R package Seurat v.4.3.0 (47). Initially, for each dataset, genes detected in fewer than three cells, cells with fewer than 500 genes, and cells with more than 25% mitochondrial gene content were excluded to ensure data quality. Subsequent normalization and scaling were performed using *NormalizeData()* function with default parameters. To identify features that capture the most significant variation in the datasets, we employed the *FindVariableFeatures()* function, selecting 2,000 highly variable genes (HVGs) for further analysis. Principal component analysis (PCA) was then applied using these 2,000 HVGs. To choose the appropriate method for data integration, we tested three batch correction tools, CCA (24), MNN (25), and Harmony (26). The *k*-nearest-neighbor batch-effect test (kBET) (23) was used to quantify batch effects and assess the performance of these tools. kBET evaluates batch mixing by testing whether the distribution of labels within a subset of neighboring samples matches that of the full dataset. It employs a chi-squared test on random neighborhoods to determine how well samples are mixed; a higher acceptance rate indicates better mixing and less batch effect. Since the Harmony demonstrated the highest performance, we applied it to adjust the principal components for removing batch effects (**Supplementary Figure S2B**). The top 10 adjusted PCs were utilized for clustering using a shared nearest-neighbor modularity optimization-based clustering algorithm, with the resolution parameter set to 2. Non-linear dimensionality reduction was conducted using t-SNE for visualization.

Following this preprocessing, cell annotation was carried out using scATOMIC (12), a modular annotation tool specifically designed for the accurate identification of malignant and non-malignant cells. scATOMIC, which was trained on over 300,000 cancer, immune, and stromal cells from 19 common cancers, employs a hierarchical approach by inputting the count matrix of our single-cell datasets into scATOMIC; we obtained detailed cell type annotations for each dataset.

### 4.3 Deconvoluting cell compositions in bulk transcriptomic data

BayesPrism (14) is a Bayesian method designed to predict cellular composition in individual cell types from bulk transcriptomic data using single-cell RNA-seq as prior information. It has demonstrated superior performance in comparison with other deconvolution tools. BayesPrism requires three inputs, the bulk transcriptomic data, raw count matrix of scRNA-seq, and cell type labels of each cell. For our analysis, only scRNA-seq dataset GSE176078 was utilized as the reference to avoid bias due to the batch effect of the integrated dataset. We then applied BayesPrism to estimate the proportions of different cell types in TNBC bulk transcriptomic data. The deconvolution was performed employing the default parameters of BayesPrism.

### 4.4 Non-negative matrix factorization

To identify subgroups of TNBC patients based on cell composition, NMF (15) was performed on the min–max normalized output of BayesPrism with each bulk RNA-seq cohort. For scRNA-seq dataset clustering, NMF was performed on the min–max normalized cell composition data obtained after scATOMIC annotation of each patient. The optimal number of clusters (rank) of NMF was determined using the cophenetic correlation coefficient.

### 4.5 Analyzing survival outcomes, differential gene expression, and functional enrichment

For the survival analysis, we utilized overall survival data from TNBC patients across three cohorts: TCGA-BRCA and METABRIC. Patients with missing survival information were excluded from the analysis. Survival curves were generated using the Kaplan–Meier method by R package survival, and differences between clusters were evaluated using the log-rank test to assess the statistical significance of survival disparities among different patient groups.

Differential gene expression analysis was performed using the R package edgeR (48). Genes were considered differentially expressed if they exhibited P-value < 0.05 and |log2Fold change| > 1. Comparing the TD and nonTD patient groups, we identified 301 differentially expressed genes (DEGs) in the METABRIC dataset and 396 DEGs in TCGA-BRCA, with 243 DEGs shared between the two datasets.

Gene Ontology (GO) analysis was conducted with the R package clusterProfiler (49) to explore the biological processes enriched among the shared DEGs. The Benjamini–Hochberg method was applied for P-value adjustment, and GO terms with P-value < 0.05 were considered significantly enriched.

### 4.6 Single-cell RNA-seq data analysis

The DEG analysis of scRNA-seq data was conducted by function *FindMarkers()* built- in R package Seurat v.4.3.0. The genes with |log2Fold change| > 1 and P-value < 0.05 are considered as DEGs. The R package irGSEA (50) was used for MHC-II pathway activity validation, and UCell was used as the scoring method. The MHC-II pathway gene set is obtained from MSigDB (30). Exhaustion score was defined as the sum of the expression of the four exhaustion markers—*ENTPD1*, *LAYN*, *ITGAE*, and *BATF* (27).

R package SuperCell (31) was used to merge cells with high similarity to metacells within the scRNA-seq data. Metacell analysis was performed for the normalized gene expression matrix by parameters with n.pc = 20, k.nn = 5, gamma = 40. After the metacell analysis, Seurat Object of metacells was created and used for subsequent analysis. Then, Harmony was used to remove the batch effect with default parameters. The unsupervised clustering of tumor cells is conducted by NMF, and optimal rank of NMF was determined based on the cophenetic correlation coefficient (**Supplementary Figure S3A**).

### 4.7 Spatial transcriptomic data analysis

R package Seurat v.4.3.0 was used for spatial transcriptomic data analysis. The pathologist’s manual labels provided in original literature were utilized as ground truth. To investigate the distribution of the MHC-II- expressing tumor cell, each tumor region spot was scored with the previously defined 40-gene signature through UCell. A higher signature score represents the higher proportion of MHC-II expressing tumor cells in each spot.

For the calculation of correlation analysis, the distance of tumor spots to the nearest lymphocyte spots obtained by calculating the minimum Euclidean distance from each tumor spot to all lymphocyte spots by the following formula:


$$\text{Euclidean Distance}=\sqrt{{\left(x_{2}-x_{1}\right)}^{2}+{\left(y_{2}-y_{1}\right)}^{2}}$$

where x_1_ and y_1_ are the coordinates of a tumor spot, x_2_ and y_2_ are the coordinates of the nearest lymphocyte spot.

The nearest Euclidean distance from the tumor spots to the nearest lymphocyte spots was divided into 10 bins, and the average of the signature score of all tumor spots in each bin was calculated for comparison.

The nearest Euclidean distance from the tumor spots to the nearest lymphocyte spots was divided into 10 bins, and the average of the signature score of all tumor spots in each bin was calculated for comparison.

### 4.8 Cell– cell communication analysis

CellChat (47) is an R package specifically developed for inferring, analyzing, and visualizing intercellular communication networks from single-cell RNA transcriptomic data. By utilizing established ligand–receptor pairings, CellChat facilitates the construction of probable communication networks among cells. For the analysis of cell–cell communication within each cellular group, a minimum cell threshold was set to 10. Communication pairs between cells were considered significant if their P-value was less than 0.05.

### 4.9 Predicting survival outcomes

To establish the prediction model, we extracted the characterize genes of MHC-II expressing tumor cells. The marker genes of MHC-II expressing tumor cells calculated from scRNA-seq DEG analysis (log2FC > 1, P_value < 0.05) and marker genes from the feature matrix returned by BayesPrism were used as the candidate genes. Then, univariable Cox regression survival analysis was performed based on the candidate genes to identify prognostic genes. Finally, 40 prognosis-related genes were selected to create a gene signature. Then, 10-fold cross-validation multivariate Cox regression analysis was performed to investigate the prognosis predictive ability of the gene signature. Next, a prognostic model was used to predict the signature score for each patient as follows:

Signature score = exp_gene1_*β_gene1_ + exp_gene2_*β_gene2_ + exp_gene3_*β_gene3_ + … + exp_gene40_*β_gene40_

where “exp” represents the gene expression, and “β” is referred to as the coefficient derived from the multivariate Cox regression analysis.

Based on the signature score equation, a signature score was obtained for each patient, and TNBC patients in each cohort could be divided into high- or low-score group using the mean signature score as the threshold. The receiver operating characteristic (ROC) curve was used to evaluate the sensitivity and specificity of the survival prediction according to the gene signature through analyzing the area under the curve (AUC) using the R package survivalROC. The defining point set up by 3-, 5-, and 7-year time-dependent ROC curve analysis was employed to assess the predictive value of the signature score for time-dependent outcomes. The Kaplan–Meier survival curve combined with a log-rank test was used to evaluate the differences in the patients’ survival time in the high- and low-score group by the R package “survival”.

### 4.10 Predicting immune infiltration

Using the gene signature from MHC-II tumor cells, we developed a random forest model to predict the immune infiltration by R package randomForest. By summing the abundance of immune cells from the bulk RNA-seq cellular composition data, the total proportion of immune cells was obtained and used as an indicator of immune infiltration. Cohort MEATBRIC was used for model training with 10-fold cross-validation, and cohort TCGA-BRCA was used as the validation set. Then, the correlation coefficient of predicated result and actual result was calculated to validate the efficacy of the immune infiltration prediction model.

## Data Availability

The original contributions presented in the study are included in the article/**Supplementary Material**. Further inquiries can be directed to the corresponding author.
