# Supplementary material for: Computational analysis of the functional impact of MHC-II-expressing triple-negative breast cancer
Source: Front Immunol. 2024 Nov 27;15:1497251. doi: 10.3389/fimmu.2024.1497251 (PMC11631845; doi:10.3389/fimmu.2024.1497251)
Supplement: Supplementary file 1 [file DataSheet1.pdf]

Supplementary material for

Computational analysis of the functional impact of MHC-II-expressing triple-negative breast cancer

Yang Cui, Weihang Zhang, Xin Zeng, Yitao Yang,
Sung-Joon Park and Kenta Nakai

Supplemental Figure 1

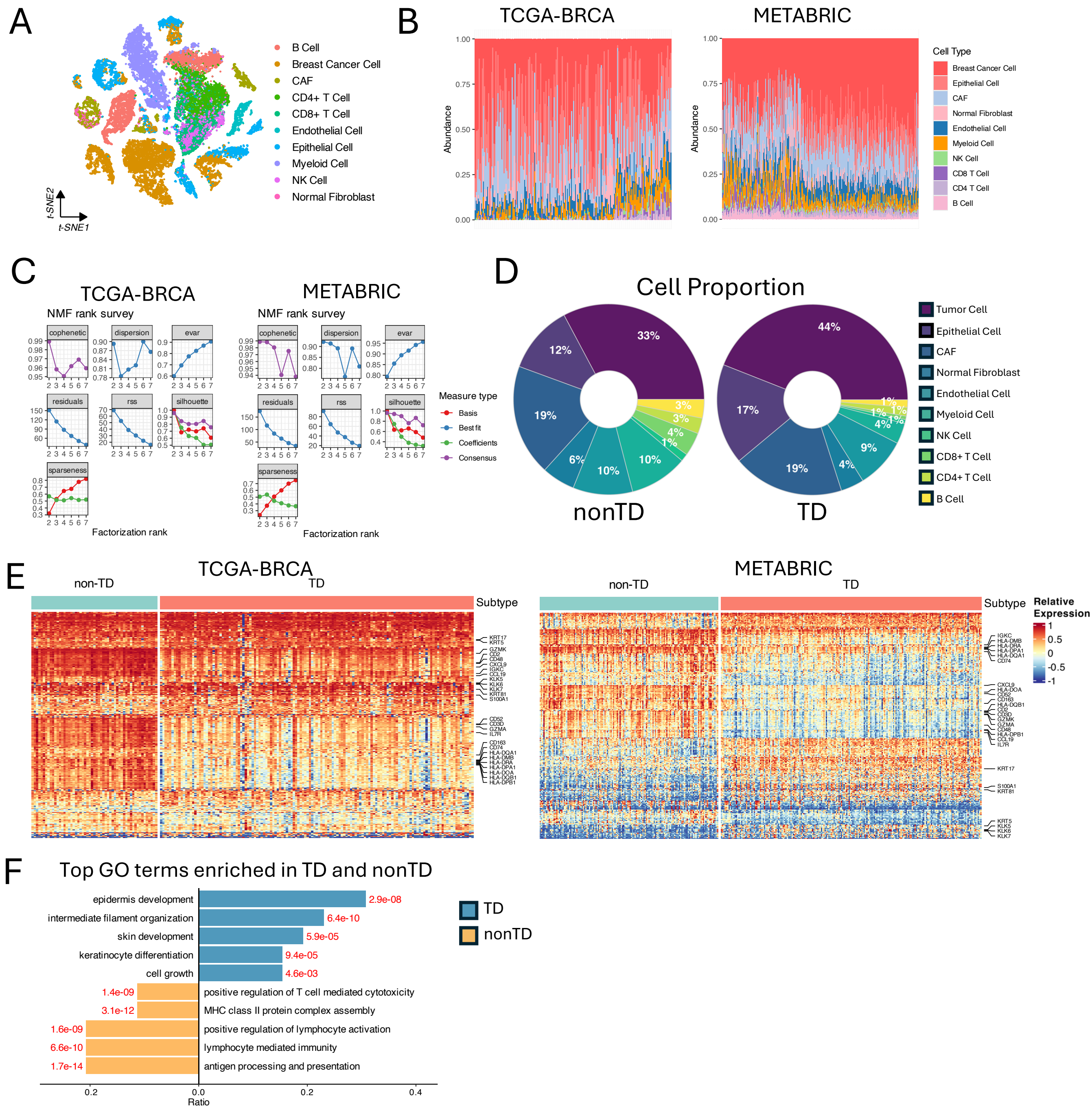

Supplemental Figure 1

A. t-SNE visualization of scRNA-seq dataset GSE176078. Cells were annotated by scATOMIC.
B. Cell proportions of TCGA-BRCA and METABRIC cohort
C. Clustering performance of NMF at different ranks.
D. Donut plots showing the cell proportion of TD and nonTD patient clusters in in the combined TCGA-BRCA and METABRIC cohort.
E. Heatmap of differentially expressed genes between TD and nonTD cluster in TCGA-BRCA and METABRIC cohort.
F. GO enrichment analysis result of biology process in TD and nonTD patient clusters. X-axis represents the gene ratio, which is the proportion of genes enriched in the analysis relative to the total number of genes associated with each biological process. The corresponding p-values for these biology process terms are displayed in red to the beside of the bars. Blue and orange bars represent the up regulated biology process in TD and nonTD, respectively.

Supplemental Figure 2

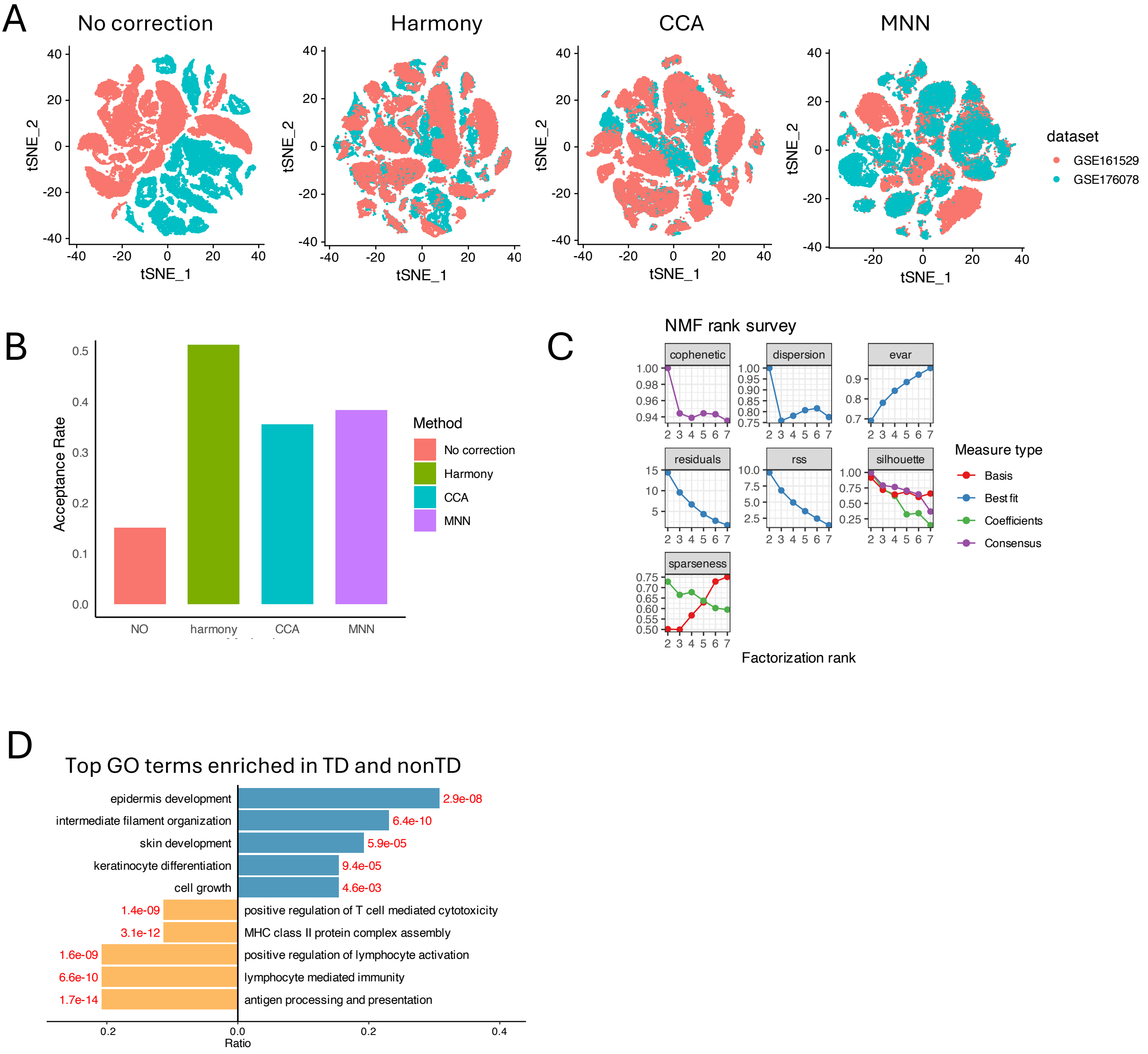

Supplemental Figure 2

E

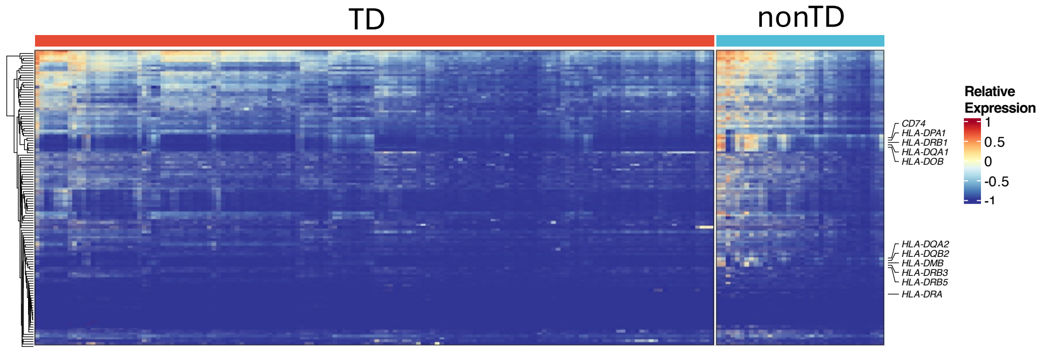

Supplemental Figure 2

A. t-SNE visualization of integrated scRNA-seq datasets (GSE161529 and GSE176078) with different batch correction tools.
B. kBET acceptance rates for dataset processed by different batch correction tools.
C. Clustering performance of NMF at different ranks.
D. GO enrichment analysis result of biology process in TD and nonTD tumor cells. X-axis represents the gene ratio, which is the proportion of genes enriched in the analysis relative to the total number of genes associated with each biological process. The corresponding p-values for these biology process terms are displayed in red to the beside of the bars. Blue and orange bars represent the up regulated biology process in TD and nonTD, respectively.
E. Heatmap showing relative expression levels of MHC-II pathway genes in TD and nonTD tumor cells.

Supplemental Figure 3

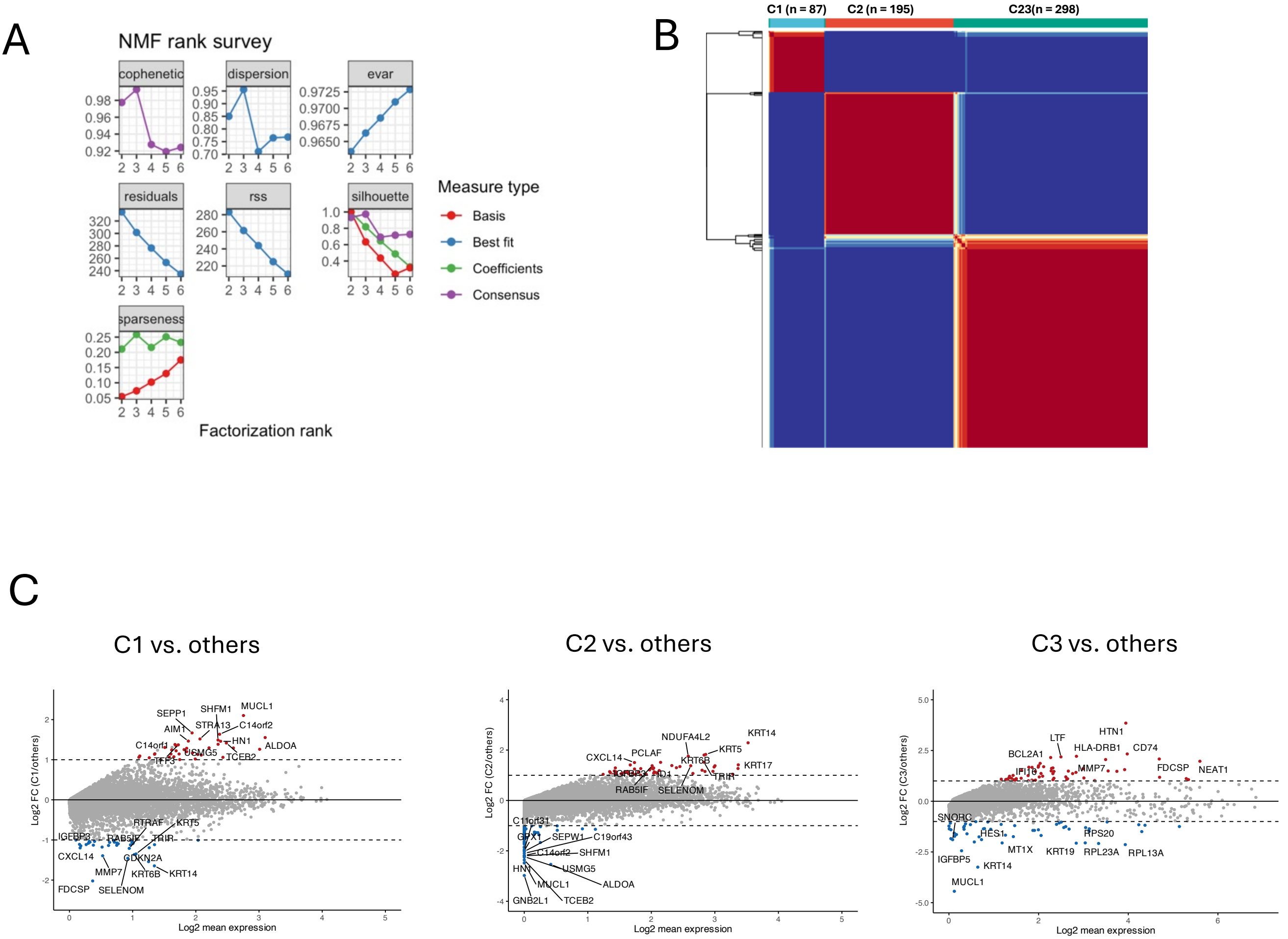

Supplemental Figure 3

D

GO enrichment of C1

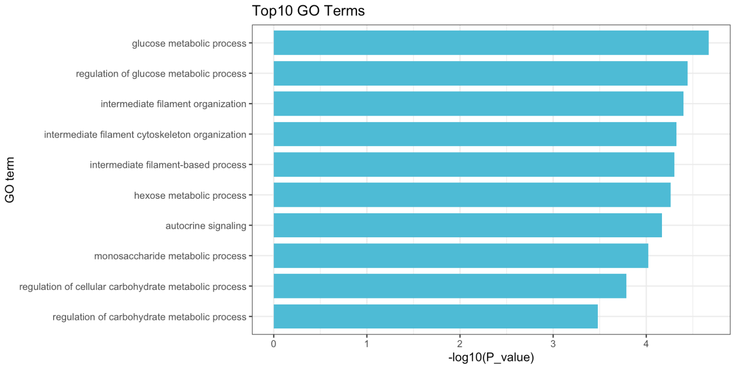

GO enrichment of C2

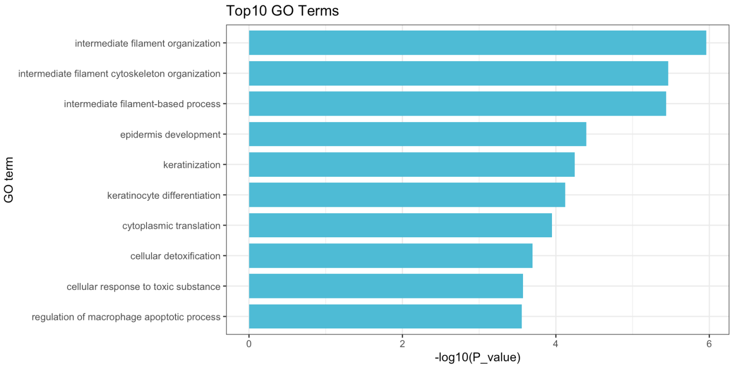

GO enrichment of C3

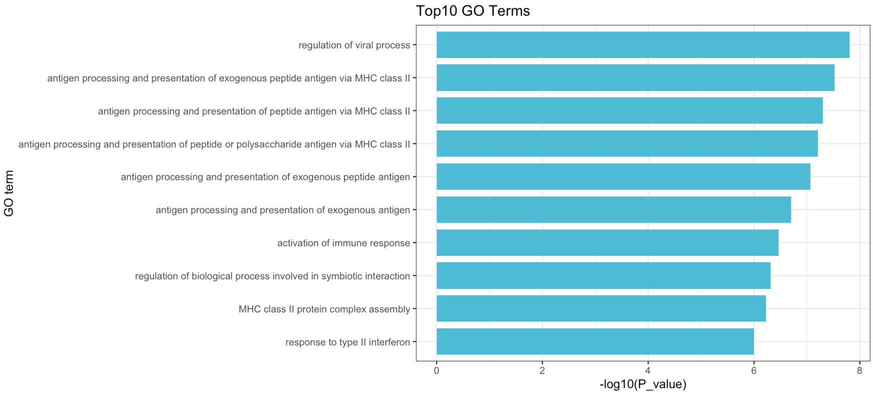

Supplemental Figure 3

E

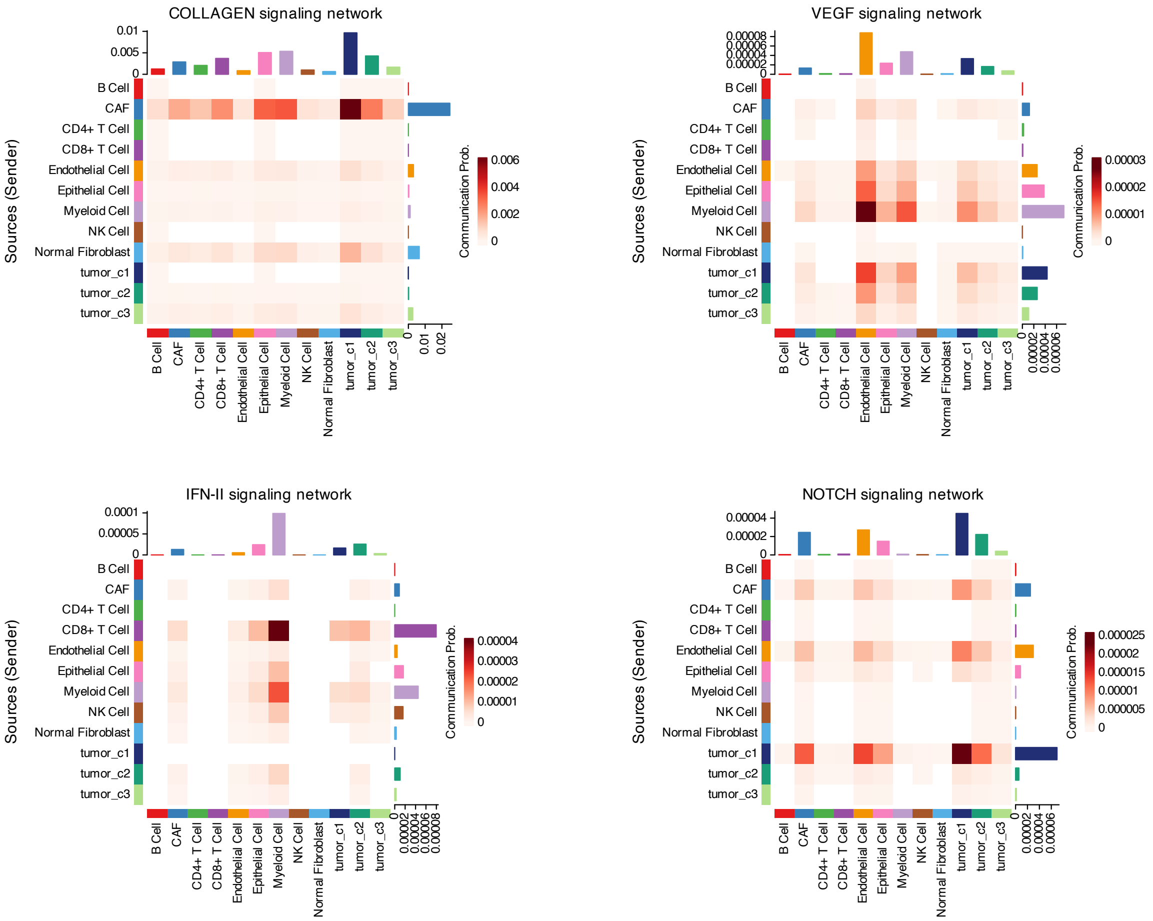

Supplemental Figure 3

A. Clustering performance of NMF at different ranks.
B. NMF clustering of metacells of tumor cells.
C. MA plot showing differentially expressed genes between c1 and other tumor cells (left), c2 and other tumor cells (middle), c3 and other tumor cells (right) (|log2FC| > 1, $p < 0.05$).
D. GO enrichment analysis result of biology process for each tumor cell subset.
E. Heatmap showing the COLLAGEN, VEGF, IFN-II and NOTCH pathway signaling interactions within the TME. X-axis represents the receiver and Y-axis represents the sender.

Supplemental Figure 4

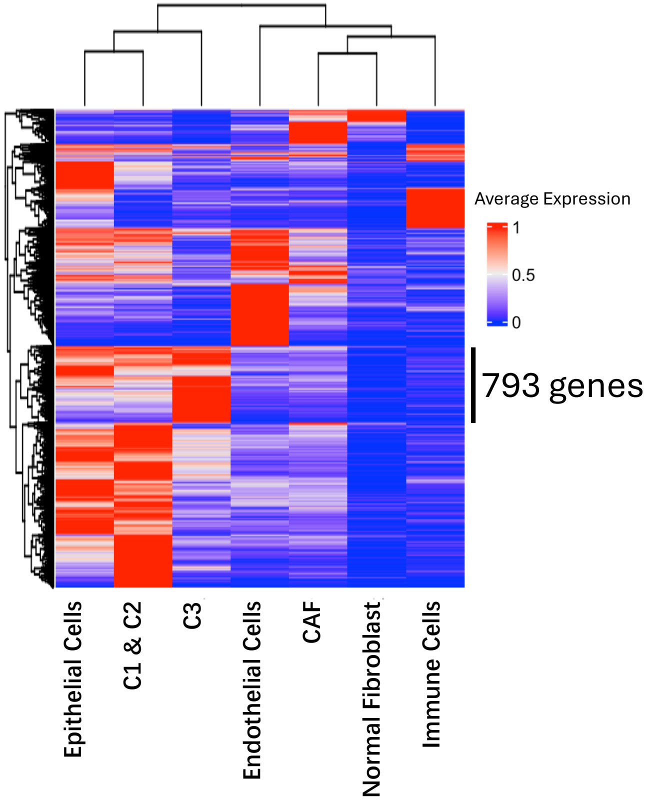

Supplemental Figure 4

Feature matrix returned by Bayesprism. Marker genes of C3 tumor cell (793 genes) were selected as part of candidate gene list for prognostic signature construction.

Supplemental Figure 5

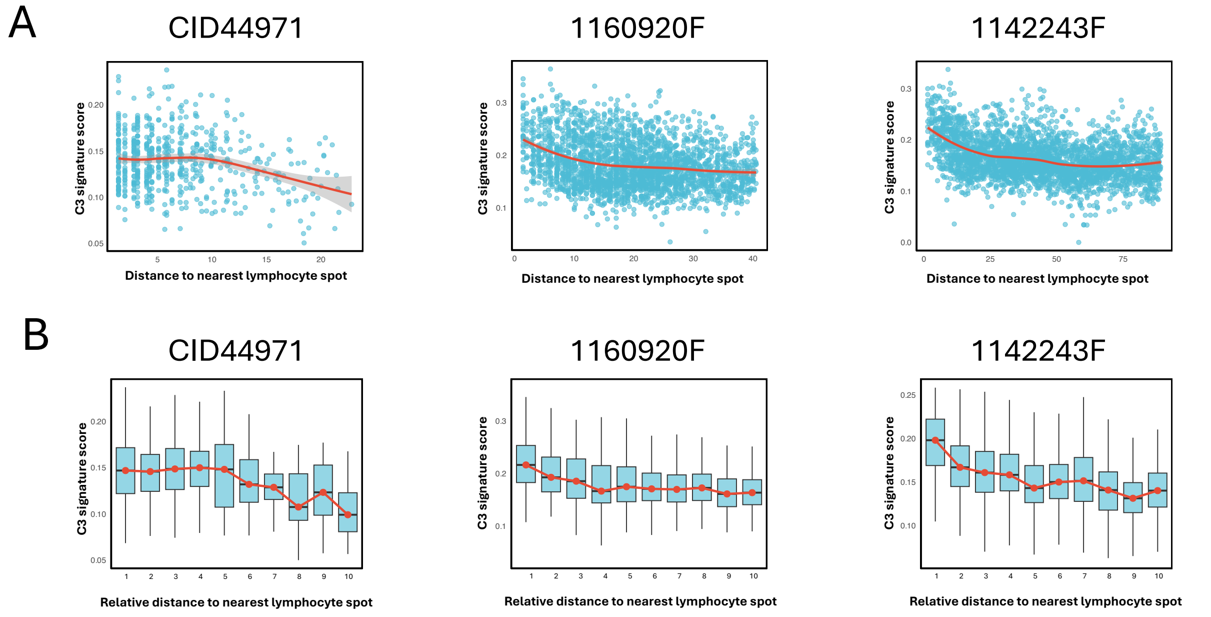

Supplemental Figure 5

A. Scatter plots showing the trend of C3 tumor cell signature score of tumor spot for the Euclidean distance to the nearest lymphocyte spot.
B. Boxplots showing C3 tumor cell signature score of tumor spots within distance bins. For each spatial transcriptomic data, tumor spots are assigned to 10 bins by their Euclidean distance to the nearest lymphocyte spot.

Supplemental Table 1

Genes	Coefficients
ARRDC2	0.08647747
BNIP3	-0.257135
BRF2	-0.3114462
DHX30	-0.585203
DIABLO	0.46530222
GBP1	-0.5909764
GBP2	0.102547
GBP4	0.19136564
GBP5	-0.1346151
GPI	0.48402126
GPX1	-0.7585415
GRWD1	0.43287137
HCLS1	0.98839541
IFI16	-0.2670963
IGHG1	-0.1107345
IGKC	0.01802405
IL27RA	-0.0331642
IRF1	-0.5316151
JCHAIN	-0.1418078
KLHL18	0.38428281
LANCL2	0.03528837
LTB	0.1122785
NME7	1.34786037
ORMDL1	-0.4198936
PKM	0.55323602
POLR2L	-0.1658288
PSMA7	0.2910336
PSPH	-0.1005668
RABIF	0.23133952
RFX5	-0.3860251
ROMO1	0.38861085
SLC2A4RG	0.3843596
SMIM15	-0.0422817
SPOCK2	-0.0613116
TMED4	-0.3090919
TMEM176A	-0.1281207
TMEM176B	0.01154591
TTC4	-0.2272285
TUBB2A	0.16252404
TUBB4B	0.32041865

Supplemental Table 1

40 genes of the prognostic signature and their coefficients.
